# Editorial: Prenatal and early life parent-child psychiatric interventions in the perinatal somatic context

**DOI:** 10.3389/fpsyt.2023.1147463

**Published:** 2023-05-15

**Authors:** Sylvie Viaux Savelon, Antoine Guedeney, Ayala Borghini, Marie-Thérèse Tauber

**Affiliations:** ^1^Hospices Civils de Lyon, Lyon, France; ^2^Institut des Sciences Cognitives Marc Jeannerod (ISC), Bron, France; ^3^Assistance Publique Hopitaux De Paris, Paris, France; ^4^Psychomotricity School HETS, HES-SO Geneva, Geneva, Switzerland; ^5^Centre Hospitalier Universitaire de Toulouse, Toulouse, France

**Keywords:** perinatality, early parent-child interaction, prematurity, early parent-child intervention, child development, emotional development

Infancy is a highly sensitive period for mental, motor, and emotional development. Such development is strongly dependent on environmental factors and particularly on the quality of early parent-child interaction.

Perinatal obstetrical and emotional factors may have effects on the first encounter with the child, thus setting the basis of parent-child interaction in a transactional perspective.

Early parent-child interaction is a dynamic, bidirectional, and synchronized process, in which the infant plays a very active part. In all likelihood, parental or child physical disorders or illnesses will have a significant impact on the ability of both the parents and child to synchronize with each other.

Recent discoveries on the biological components of mother-fetus and mother-child synchrony have given new insights into the links between parent-infant interactions and children's neurological and emotional development. Medical advances in obstetrics as well as in neonatal intensive care have opened up the possibility to investigate these questions in new populations of infants and parents who had not been previously studied, for example, very early premature babies at 24 weeks gestational age, children operated *in utero* at birth for cardiac defects, or severely disabled mothers who had no access to maternity care.

All these advances have allowed for much more favorable outcomes for the infants in question than could have been imagined before. This medical progress has opened up a whole new field for child mental health and psychiatry, with a series of new ethical issues needing to be addressed. Early perinatal parent-child interventions may be highly useful for infants born in these situations but studies are still rare in this new field. To guide clinical practice and treatment, further research is needed.

The objectives of this Research Topic are to present data and studies on perinatal and early life parent-child interactions from the prepartum period up to 2 years of age (i.e., preverbal children) based on studies on families in these specific circumstances.

The majority of the articles on this Research Topic deal with premature babies, their parents, and their relationship, focusing on a series of different questions: symptoms and assessment scales (Boissel et al.; Dollberg et al.), specific treatment approaches (Boissel et al.; Bustamante Loyola et al., Vitte et al.), prenatal exposition to drugs (Benevent et al.), prenatal stress (1), cleft lip and palate (Pérez Martínez et al.), and early symptom formation (2).

Maternal sensitivity is affected by prematurity. Scales seeking to measure parent-premature baby interactions are rare. The development of early psychotherapy to foster maternal sensitivity during children's hospitalization in neonatal intensive care units (NICU) is a major challenge for the prevention of subsequent developmental and mental disorders in the child. Boissel et al. from Amiens in France present a new tool exploring early interactions for premature babies in their paper entitled “*Development and reliability of the coding system evaluating maternal sensitivity to social interactions with 34- to 36-week postmenstrual age preterm infants*.” The team developed a procedure for assessing maternal sensitivity in interactions between the mothers and their 34- to 36-week preterm infants. The resulting Preterm Infant Coding System for Maternal Sensitivity (PRICOSMAS) presents good internal consistency and interrater reliability and could therefore be a useful tool for both care professionals and researchers.

Dollberg et al., from Israël, analyzes the interaction between premature babies and their parents through the prism of parental reflective functioning and its influence on parenting stress. Her article “*Parental reflective functioning as a moderator of the link between prematurity and parental stress*” helps us to better understand this aspect of parent-child interactions, with perspectives to help alleviate the parent's burden.

During their first year, preterm infants have a higher probability of developing sustained social withdrawal than infants born at full term. Bustamante Loyola et al., from Santiago in Chile, describes in his article “*The impact of an interactive guidance intervention on sustained social withdrawal in preterm infants in Chile: randomized controlled trial*” the efficacy of a standardized behavioral intervention by pediatricians on sustained social withdrawal in preterm infants. He shows how this intervention reduces the prevalence and the possible associated negative outcomes of children's withdrawal behavior.

Boissel et al.'s study, “*A narrative review of the effect of parent-child shared reading in preterm infants*,” shows the positive effect of shared reading sessions on the physiological parameters of preterm infants in neonatal intensive care. An original therapeutic mediation—book-reading to a premature child in intensive care—was tested in a neonatal intensive care unit. The team proposed to the parents to read out loud to their premature children in the NICU. This therapeutic mediation was found to be feasible and well-accepted as it provided concrete support for positive parenting in this highly stressful context.

Vitte et al., from France, created with her team an innovative mother-baby unit integrated into a neonatal care service. She describes this new unit and its function in her article: “*Panda unit, a mother-baby unit nested in a neonatal care service*.”

Strategies for handling other child pathologies that impair early interactions are also explored. Cleft lip and palate, for example, clearly impose a higher risk of physical and emotional distress in infants with a major impact on parent-infant relationships. Pérez Martínez et al. in her article “*The prevalence of social withdrawal in infants with Cleft Lip and Palate: the feasibility of the full and the modified versions of the Alarm Distress Baby Scale (ADBB)*” shows the interest in the ADBB scale for evaluating social withdrawal as a sign of distress in these infants. Indeed, her study found a relatively high level of social withdrawal in infants with cleft lip and palate but with a higher prevalence in “simple” lip cleft compared to cleft lip associated with palate clefts.

The epidemiological study developed by Benevent et al.'s team, from France, in her article “*Prenatal drug exposure in children with a history of neuropsychiatric care: a nested case-control study*” highlights links between children's neuropsychiatric symptoms and their exposure to nervous system drugs *in utero*. She provides guidelines for assessing the long-term neuropsychiatric effects after prenatal medication exposure, without focusing on psychotropic medications.

Encouraging research in this new field of perinatality, a transversal and transdisciplinary field at the crossing of obstetrics and pediatrics, as well as child and adult psychiatry is clearly a key issue. All of the present articles will hopefully lay the ground for a whole new area of study to be developed over the coming years.

## Author contributions

SV wrote the first draft of the manuscript. AG, AB, and MT-T contributed to manuscript revision, read, and approved the submitted version.
